# Genotyping Oral Commensal Bacteria to Predict Social Contact and Structure

**DOI:** 10.1371/journal.pone.0160201

**Published:** 2016-09-29

**Authors:** Stephen Starko Francis, Mateusz M. Plucinski, Amelia D. Wallace, Lee W. Riley

**Affiliations:** 1 Division of Epidemiology, School of Public Health, University of California, Berkeley, California, United States of America; 2 Division of Epidemiology and Biostatistics, University of California, San Francisco, California, United States of America; 3 Department of Environmental Science and Policy Management, University of California, Berkeley, California, United States of America; 4 Division of Infectious Diseases and Vaccinology, School of Public Health, University of California, Berkeley, California, United States of America; Medical University of South Carolina, UNITED STATES

## Abstract

Social network structure is a fundamental determinant of human health, from infectious to chronic diseases. However, quantitative and unbiased approaches to measuring social network structure are lacking. We hypothesized that genetic relatedness of oral commensal bacteria could be used to infer social contact between humans, just as genetic relatedness of pathogens can be used to determine transmission chains of pathogens. We used a traditional, questionnaire survey-based method to characterize the contact network of the School of Public Health at a large research university. We then collected saliva from a subset of individuals to analyze their oral microflora using a modified deep sequencing multilocus sequence typing (MLST) procedure. We examined micro-evolutionary changes in the *S*. *viridans* group to uncover transmission patterns reflecting social network structure. We amplified seven housekeeping gene loci from the *Streptococcus viridans* group, a group of ubiquitous commensal bacteria, and sequenced the PCR products using next-generation sequencing. By comparing the generated *S*. *viridans* reads between pairs of individuals, we reconstructed the social network of the sampled individuals and compared it to the network derived from the questionnaire survey-based method. The genetic relatedness significantly (p-value < 0.001) correlated with social distance in the questionnaire-based network, and the reconstructed network closely matched the network derived from the questionnaire survey-based method. Oral commensal bacterial are thus likely transmitted through routine physical contact or shared environment. Their genetic relatedness can be used to represent a combination of social contact and shared physical space, therefore reconstructing networks of contact. This study provides the first step in developing a method to measure direct social contact based on commensal organism genotyping, potentially capable of unmasking hidden social networks that contribute to pathogen transmission.

## Introduction

Since the mid 1970’s when Cassel, Cobb, Berkman and Syme [1–3] first showed that social networks influence health, social connectivity has been increasingly recognized as an essential determinant of disease. How individuals form groups and interact is directly linked to the distribution of diseases in the population [1, 4, 5], a fact highlighted by the recent Ebola epidemic and reemergence of vaccine preventable diseases in the United States. Notably, this applies not only to infectious diseases, where the spread of diseases is sensitive to the structure of the contact network [6], but also to chronic diseases such as obesity, where individuals share common exposures [7].

Unfortunately, the importance of knowing the structure of social networks is matched by the difficulty in accurately measuring them, and an empirical measure of social distance remains elusive. Traditional methods, such as questionnaire-based surveys, are costly, time-intensive, and inaccurate. They are also limited in their ability to decipher cryptic transmission events associated with chronic infections, such as tuberculosis caused by *Mycobacterium tuberculosis* and AIDS caused by HIV. As a result of shortcomings to traditional methods, there has been increasing attention on creative ways of measuring social networks, such as using radio-frequency identification tags to measure proximity between individuals [8].

Here, we tested the hypothesis that the contact network structure, defined as physical contact and shared environment, of a population can be characterized based on the microevolution and transmission of commensal bacteria. During outbreak investigations, transmission networks are routinely reconstructed by genotyping and comparing etiologic isolates from infected individuals [9–14]. Analogously, outside the context of outbreaks, contact networks might be able to be reconstructed solely by genotyping and comparing commensal bacterial isolates from individuals in the network [15]. Such an approach may allow investigators to track chains of transmission more accurately and quickly during epidemics, and unlike questionnaire-based assessment, could reveal physical contact that may be unknown to the subjects. As an analogy to traditional multi-locus sequence typing (MLST) of bacterial strains, we call our method metaMLST. Whereas MLST is based on culturing followed by Sanger sequencing of housekeeping genes of a single strain at a time, our method is specifically designed to amplify and identify sequences from multiple strains in microflora simultaneously via next-generation sequencing. In contrast to traditional metagenomic approaches, our method does not examine broad microbiome structure but rather targets nucleotide differences across *Streptococcus viridans* at diverse MLST sites to detail micro-evolutionary changes in the bacteria, that allows for reconstruction of transmission events.

## Materials and Methods

### Study Design

The study population consisted of current faculty and staff within the School of Public Health (SPH) at a large research university. An initial recruitment email was sent out to all SPH faculty and staff (*n = 430*) containing a link to a brief online questionnaire asking about the individual’s top five social contacts within the SPH. Seventy-two (17%) individuals filled out the first questionnaire and of these, 52 (72%) individuals were enrolled as study participants, and eight nominated their spouse or domestic partner to also participate. Study participants provided saliva samples and filled out questionnaires regarding factors potentially influencing the oral microbiome and their social contacts within the SPH.

The study was approved by the UC Berkeley Committee for Protection of Human Subjects. Each study subject was required to read, understand and sign an informed consent document detailing the study and use of data.

### Study Questionnaire

Of the 60 participants, 59 completed a detailed questionnaire documenting antibiotic use, smoking, and oral hygiene. They also provided their top twenty contacts in the SPH and, for each contact, estimated the number of hours per week of face-to-face contact.

### Sample Collection

Saliva samples were collected from 52 faculty and staff and 8 domestic partners/spouses. Sample collections were conducted during a nine-day period in March 2012. Five milliliters of saliva were collected from each participant into a sterile 50mL tube and delivered on ice immediately to a campus laboratory where the saliva was separated into 1 mL aliquots in sterile, nucleic acid-free tubes and stored at -80°C. All laboratory procedures were performed with care to maintain sterile, nucleic acid-free conditions. Fifty-five subjects with suitable material were included in the study.

### DNA Extraction and PCR

The Qiagen DNeasy Blood and Tissue Kit (Qiagen, Valencia, CA) was used according to manufacturer’s instructions to extract DNA from 1 mL of saliva following the pre-treatment protocol for Gram positive bacteria. Amplification of 8 housekeeping genes of viridans group *Streptococcus* using degenerate primers was performed according to a previously established protocol [16]. Briefly, 2 μl extracted template DNA was used in a 50 μl PCR reaction mixture containing 0.2 mM dNTPs, 1x PCR Buffer II (Invitrogen, Carlsbad, CA), 1.5 uM MgCl, 1.0 μM each forward and reverse primer, and 2.5 U Amplitaq Gold DNA Polymerase (Invitrogen, Carlsbad, CA) for reactions with primers for *ppaC*, *pyk*, *tuf*, and *map*, while reactions with primers for *pfl*, *guaA*, *soda*, and *rpoB* received 1.5 U. PCR conditions followed the previously-published protocol.

For each study subject, 10 μl of PCR product from each of the 8 housekeeping genes was pooled and purified with the Qiagen QIAquick PCR Purification Kit (Qiagen, Valencia, CA) according to manufacturer’s instructions. Final product concentrations were assessed with a Nanodrop ND-1000 (Nano-Drop Technologies, Wilmington, DE) spectrophotometer, and diluted in sterile water to ~1-2ng/μl in 120 μl final volume. Each sample was barcoded and prepped individually using PrepX ILM DNA Library kits (WaferGen, Fremont, CA) according to the manufacturer’s protocol. The library was PCR amplified and the reaction was cleaned with AGENCOURT® AMPURE® XP (Beckman Coulter, Brea, CA, USA). The samples were sequenced using the Illumina HiSeq2000 platform with 100bp paired end sequencing (Illumina, Inc, San Diego, CA). Amplified DNA from twelve participants and three controls was first run on a single Illumina lane, and DNA from the remaining 48 participants was subsequently run on a second Illumina lane.

### Social Network Analysis

**Fig 1:**
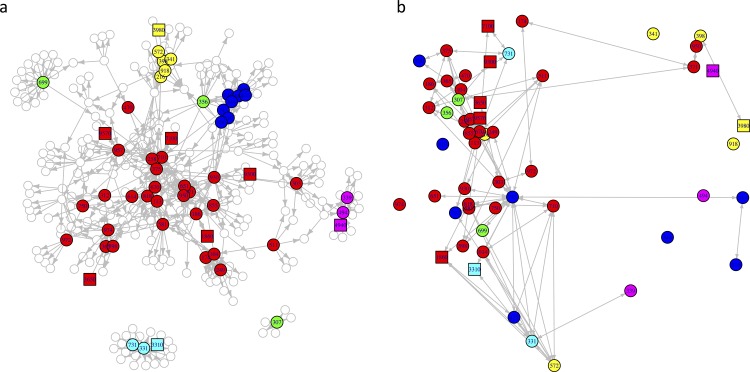
Networks. a) Questionnaire-based network from the SPH faculty and staff and B) network reconstructed from genetic data from oral commensal bacteria. Nodes are colored by campus building locations of offices. Circular nodes represent faculty and staff, and square nodes represent spouses.

A social network was created from the data collected on both the primary, screening questionnaire and the secondary detailed questionnaire for study participants. The network included 59 full study participants and 12 faculty and staff that filled out the screening questionnaire but were not enrolled (Fig 1). Two nodes were considered to be linked if one of the nodes listed the other as a contact. The degrees of separation, defined as the length of the shortest path between two nodes, were calculated for each pair of individuals. The average hours per week of face-to-face contact time were calculated for pairs of individuals where at least one individual had filled out the screening questionnaire.

### Processing of Sequencing Results and Reconstruction of Network

Approximately 20 million reads were generated per subject from the Illumina runs. Data were filtered for quality based on an extremely strict filter, where every base pair was required to have a phred score of 35 or greater or the entire 100bp read was excluded. Approximately 60% of raw reads did not meet the quality filter, and data from a total of 55 subjects were analyzed. Quality filtered raw sequencing data were added to the NCBI Short Read Archive (S1 File).

An index of unique 100bp reads was created by pooling all high quality reads from all subjects, then removing the duplicate reads, both forward and reverse reads were used. A Bowtie2 [17] index of the 14,997,671 unique reads was created and each subjects’ quality filtered reads were aligned to this index by Bowtie2, and pairwise comparisons resulted in 1485 unique comparisons. Each of these unique comparisons resulted in an alignment to the unique read index. Each of the resulting SAM files were parsed by Samtools [18] and imported to R version 3.0.1 (R Foundation for Statistical Computing, Vienna, Austria) for analysis.

Genetic relatedness was calculated by summing the number of shared unique reads for each pair of individuals and then dividing by the total number of unique reads for both individuals. The distribution of genetic relatedness was plotted, stratified by pairs of contacts with network distance >2, second order contacts, non-spousal first order contacts, and spousal contacts. The distributions were compared using the Kolmogorov–Smirnov test.

All possible pairs of individuals were sorted by genetic relatedness, and the top 79 (the number of links in the observed network) pairs of individuals were considered to be linked in the reconstructed network. The full set of unique reads was subsampled 1000 times, and for each pair of individuals, the probability that the reconstructed network included a link between the pair of individuals was calculated. The probability that spousal contacts, non-spousal first order contacts, and all first order contacts combined would be linked was calculated and compared to the null probability of any two pairs being linked. This null probability was calculated as the number of links in the observed network divided by the total number of all possible pairs in the network. The 95% confidence intervals were calculated assuming a binomial distribution.

## Results

Seventy one staff and faculty of the SPH distributed over six different campus building locations completed questionnaires where they declared which SPH faculty and staff they had face-to-face contact with on a regular basis. The network constructed on the basis of the declared contacts showed a contact structure that clustered by campus location (Fig 1A). A subset of 52 faculty and staff, and 8 spouses of faculty and staff provided saliva samples and completed a more detailed questionnaire where they reported the average hours of face-to-face contact per week with all declared contacts among all SPH faculty and staff.

**Fig 2:**
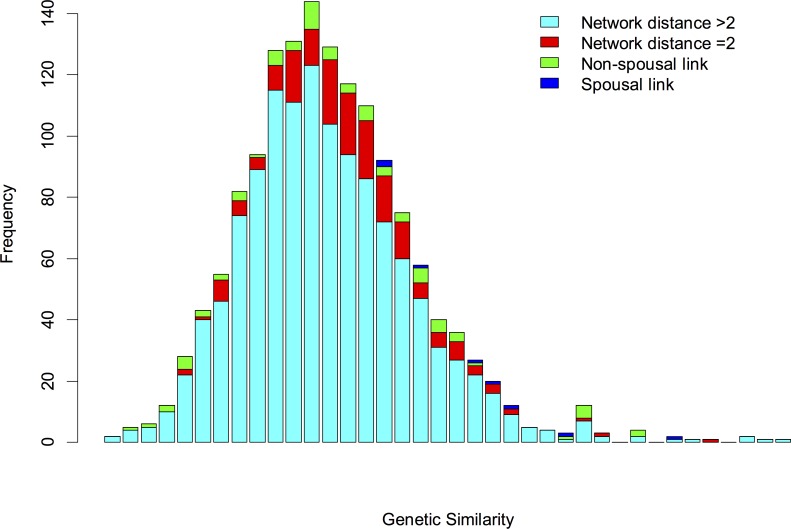
Histogram of genetic relatedness between pairs of isolates. Links between declared contacts (blue and green) are overrepresented in the long upper tail of the distribution.

**Fig 3:**
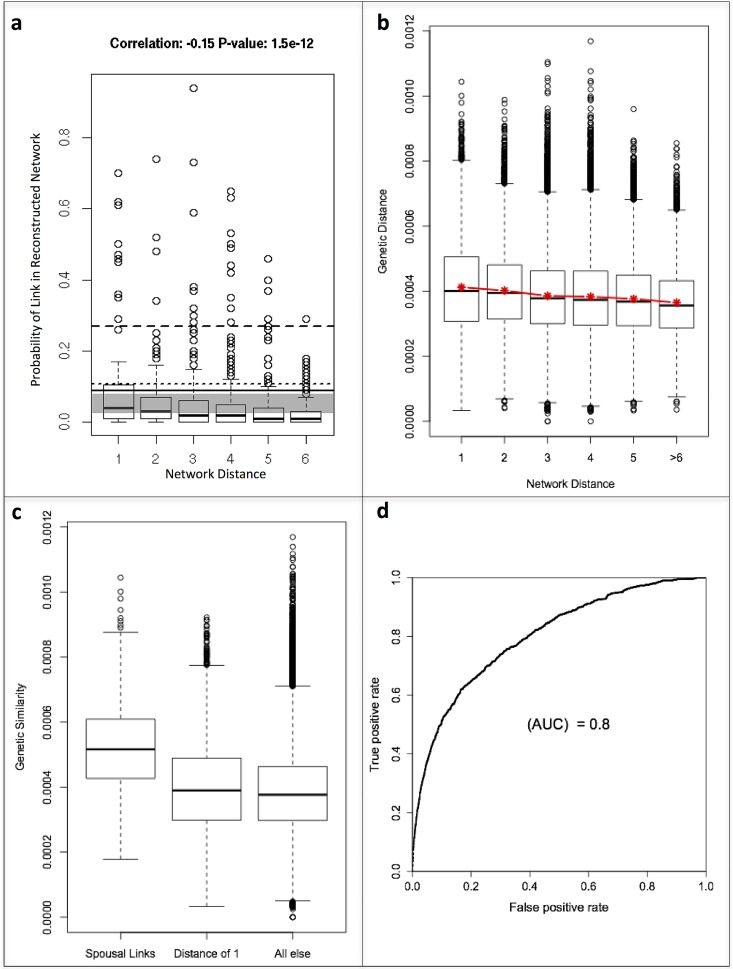
Network Relatedness. a). Boxplots show the distribution of genetic relatedness between spousal links, non-spousal declared links, and links with no reported contact. b) Boxplots show the genetic relatedness between pairs of isolates as a function of the network distance, defined as degrees of separation, in the questionnaire-based network. Red line and stars show mean and trend. c) Boxplots show the probability of two individuals being linked in the reconstructed network as a function of the network distance of the individuals in the questionnaire-based network. The gray area represents the range of probabilities expected by chance; probabilities above this area are links over-represented in the reconstructed network and probabilities below are links under-represented in the reconstructed network. The dashed line is the average probability of a spousal contact being identified as a link in the reconstructed network; the dotted line is the average probability of a non-spousal declared contact being identified as a link in the reconstructed network; and the solid line is the average probability of a declared contact in the observed network being identified as a link in the reconstructed network. d) Receiver operating characteristic (ROC) curve representing the predictive power of differentiating spouses from non-spouses using genetic relatedness between pairs of isolates.

Sufficient amounts of *Streptococcus viridans* DNA were amplified from saliva samples from 55/60 (92%) sampled individuals. The topology of the reconstructed contact network inferred from the *Streptococcus viridans* genotyping of the 55 individuals followed a similar structure to the questionnaire-based network (Fig 1B) and correctly identified 9.0% of all links, versus 5.3% expected by chance (p-value < 0.001). The genetic relatedness of the amplified *Streptococcus viridans* fragments between all pairs of sampled individuals (n = 1485) followed a skewed distribution with a long upper tail (Fig 2). The distribution of genetic relatedness was significantly different between pairs of individuals with network distance >2 (more than 2 degrees of separation apart) and second-order contacts (Kolmogorov-Smirnov p-value <0.01), non-spousal first-order contacts (p-value <0.001) and spousal contacts (p-value < 0.0001). Links reported among the 55 sampled individuals in the questionnaire-based network were over-represented (p-value < 0.001) in the top links as sorted by genetic relatedness. The probability of a pair of nodes being linked in the reconstructed network was significantly correlated (p-value < 0.001) with network distance, defined as degrees of separation, in the questionnaire-based network (Fig 3A), and genetic relatedness declined with increasing network distance in the questionnaire-based network (Fig 3B). The average probability that spousal contacts, non-spousal first-order contacts, and all first-order contacts combined would be linked in the reconstructed network was higher than that expected at random. Genetic relatedness was highest between spouses, followed by declared non-spousal first order contacts (Fig 3B). Predictability of spousal contacts was highest, with a receiver operating characteristic (ROC) area under the curve of 0.80 (Fig 3D).

## Discussion

Our results provide evidence that oral commensal bacteria are shared between individuals with routine social contact and that genetic relatedness between individuals' oral microbiomes varies with their social distance. Unlike traditional microbiome analyses which for broadly examine coarse, genus-level microbiome structure, our technique examines micro-evolutionary changes in *S*. *viridans* enabling high-resolution examination of recent transmission events. Like previous studies of *E*. *coli* distribution in animal populations [19], we show that genetic relatedness of bacteria between humans in a population can be used to reconstruct the social network structure of the population. However we cannot completely de-convolute the role that shared environment plays in our reconstruction where physical objects likely serve as a vehicle for *S*. *viridans* transmission alongside direct physical contact. This is despite the fact that the questionnaire-based network used as the “gold-standard” here is itself subject to many limitations, and is an imperfect representation of the true contact network. We are possibly detecting recent transmission events that were not captured in the survey or were unknown to the research subjects; these events may range from shared surfaces (fomites) and similar diet to direct contact with unreported subjects. Ultimately, there is no single definition of a contact network, with the ‘true’ network for comparison depending on the research question. Finally, we are also not able to rule out shared risk factors and common environmental exposures leading to genetic relatedness as a potential alternative hypothesis for high genetic relatedness amongst social contacts.

The use of genotyping techniques to investigate contact network structure could have multiple potential applications. Traditionally, assessment of social networks has been mainly qualitative, difficult to measure, and rife with error [20, 21]. Using metaMLST, a direct and quantitative measure of the social contact network in an outbreak scenario or in a long-term cohort would provide essential information on the social structure that may be a fundamental determinant of the disease. An additional advantage is the ability to quantify social contact that is both known and unknown to the subject. Furthermore the principle of a meta-MLST based system could be implemented at a very low cost compared to broad untargeted metagenomic analyses [22]. If optimized in a low cost form meta-MLST may allow both public health and academic utilization on a large scale.

The scope of diseases that may benefit from an accurate, reliable, and valid measure of social distance is broad: ranging from infectious diseases, to chronic diseases, to conditions of unknown etiology. The relatedness of oral microbes could be used to predict how epidemics would spread through a population. The recent Ebola epidemic highlights the need for a rapid and inexpensive tool to define social contact to efficiently cohort and monitor exposed individuals [23]. On the other hand, the observation that commensal bacteria are likely transmitted through social contact provides a potential explanation for why certain chronic diseases cluster in populations. Traditional epidemiologic measures coupled with meta-MLST may enhance our understanding of common conditions such as obesity, cardiovascular disease, cancer, stroke, diabetes, and asthma by providing a direct measure of social contact. This would allow researchers to systematically characterize important factors such as neighborhood effects, particularly important in the study of health disparities. The concept of neighborhood may be refined based on the rate of uptake of bacteria from social sources [24], combining the social and spatial concept of neighborhood with direct measurements of social contact from metaMLST to refine the assessment of social interactions. These data could in turn generate novel theories regarding the complex web of causation of disease [25, 26].

## Supporting Information

S1 File SRA accession numbers. (TXT)
